# Mad Honey Poisoning Case Series Presenting With ECG Findings Including Atrioventricular Complete Block and Symptomatic Hypotension

**DOI:** 10.7759/cureus.17165

**Published:** 2021-08-13

**Authors:** Mehmet Sami Islamoglu, Mehmet Dokur, Kanan Talibli, Betul Borku Uysal, Emrah Ozdemir

**Affiliations:** 1 Department of Internal Medicine, Biruni University Medical Faculty, İstanbul, TUR; 2 Department of Emergency Medicine, Biruni University Medical Faculty, İstanbul, TUR; 3 Department of Cardiology, Biruni University Medical Faculty, İstanbul, TUR

**Keywords:** mad honey, atrioventricular complete block, grayanotoxin, bradycardia, rhododendron

## Abstract

Plants belonging to the Ericaceae family, which grow endemically in some parts of the world, contain grayanotoxin, which causes fatal bradyarrhythmia and circulatory collapse. Mad honey, which comes from plants with grayanotoxin, has various uses, namely, as an aphrodisiac, as an alternative therapy for GI disorders such as peptic ulcer, dyspepsia, and gastritis, and as a treatment for hypertension. However, GI, neurological and cardiac side effects may arise from its ingestion due to the grayanotoxin contained by this type of honey. Cardiac rhythm disturbances, sinus bradycardia, and other life-threatening side effects can occur, especially atrioventricular (AV) block and nodal rhythms. In this article, we present five honey poisoning cases involving adults who were admitted to our ED. Notably, one of the patients was unresponsive to atropine, so a temporary pacemaker was inserted, after which the patient was moved to the coronary ICU. Meanwhile, the cardiac rhythm of the other cases returned to normal in the follow-up after atropine administration.

## Introduction

Every year, incidents of honey poisoning occur all over the world due to a type of honey called ‘mad honey' in Turkey [1]. The reason for the poisoning is a compound called grayanotoxin, which can be found in the pollens, nectars, flowers, and leaves of numerous variants of rhododendron plants in the Ericaceae family [2]. Grayanotoxin affects voltage-gated sodium channels and mostly causes symptoms like hypotension, rhythm disorders like atrioventricular (AV) block bradycardia, nausea, vomiting, sweating, exhaustion, and mental fog [3]. In this case series, we present five cases of mad honey poisoning admitted to our ER and briefly discuss the related literature.

## Case presentation

**Figure 1:**
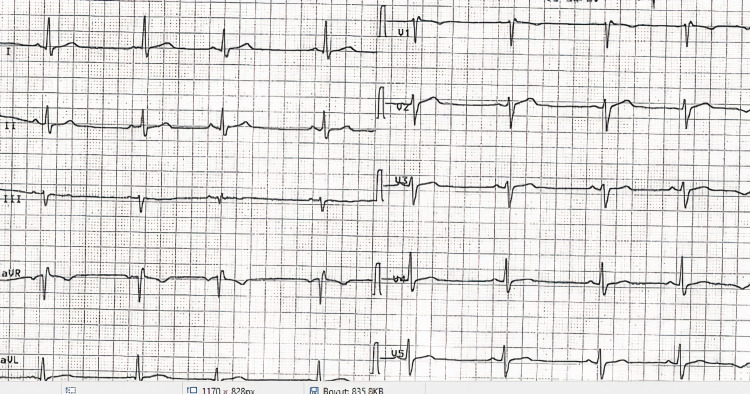
Atrioventricular complete block.

**Figure 2:**
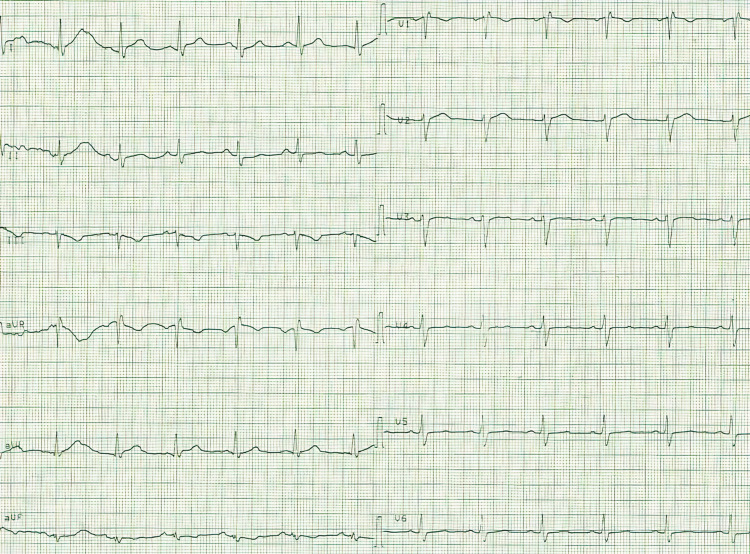
Sinus rhythm after temporary pacemaker.

Case 1: A 50-year-old male patient was admitted for emergency service with symptoms of hypotension, dizziness, and bradycardia due to honey poisoning. The patient was hypotensive (blood pressure: 80/50 mm/hg), his heart rate was 30 beats per minute, his body temperature was 36°C and his SpO2 was 98. The ECG findings were fully indicative of bradycardia and AV complete block (Figure 1). The laboratory findings were as follows: 22 mg/dL urea, 1 mg/dL creatinine, 3.6 mmol/L potassium, 141 mmol/L sodium, 13 U/L aspartate aminotransferase (AST), 17 U/L alanine aminotransferase (ALT), 13400 K/uL leukocytes, 14.6 g/dL hemoglobin, 322000 K/uL platelet count, 2 mg/L C-reactive protein (CRP), 10.30 U/L creatine kinase-MB (CK-MB), 1.6 pg/mL troponin, and 700 ng/mL D-dimer. Since the pulse of the patient was 30 beats per minute on the emergency service and diagnosis of AV complete block was made, our team transferred him to emergency angiography. A temporary pacemaker was implanted in the patient. Thereafter, the patient’s condition became stable in the general ICU. He was discharged from the coronary ICU after his heart rhythm reverted to normal (Figure 2).

Case 2: A 60-year-old male patient was admitted to our service with symptoms of chest pain, hypotension, and bradycardia. He was transferred to the coronary ICU due to the following: 120/70 tension, pulse of 55 beats per minute, 36°C body temperature, and 98 SpO2. The laboratory findings were 140 mmol/L sodium, 4 mmol/L potassium, 27 mg/dL urea, 1.36 mg/dL creatinine, 20 U/L AST, 37 U/L ALT, 5 mg/L CRP, 11 U/L CK-MB, 1.6 pg/mL troponin, 386 ng/mL D-dimer, 8300 K/uL leukocytes, 15.6 g/dL hemoglobin, and 267000 K/uL platelet count. The patient’s pulse reached 55 beats per minute following the IV administration of atropine. He was discharged after undergoing monitoring for a certain period at the coronary ICU.

**Figure 3:**
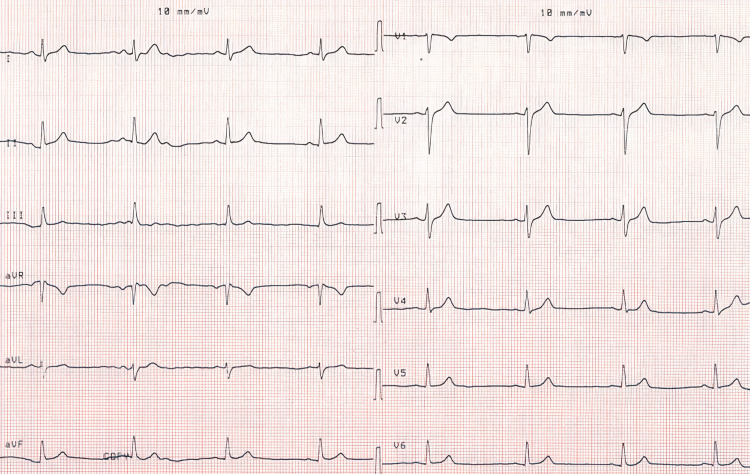
Sinus bradycardia before treatment.

**Figure 4:**
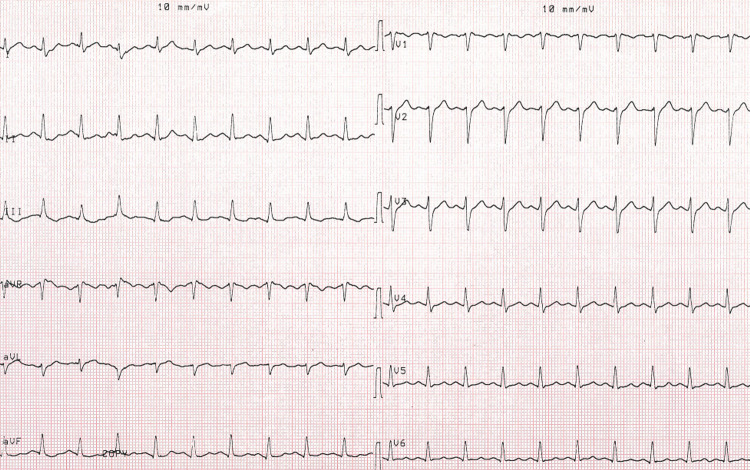
Sinus rhythm after IV administration of atropine.

Case 3: A 46-year-old male patient was taken to the hospital in an ambulance because he was suffering from senselessness and numbness. The pulse of the patient was 47 beats per minute when he arrived at the ER. He was evaluated by the cardiology unit and monitored. The ECG findings were indicative of sinus bradycardia (Figure 3). The laboratory findings were 23 mg/dL urea, 0.9 mg/dL creatinine, 140 mmol/L sodium, 4.3 mmol/L potassium, 7500 K/uL leukocytes, 13 g/dL hemoglobin, 224000 K/uL platelet, 2 mg/L CRP, 25.8 U/L CK-MB, 16 U/L AST, 15 U/L ALT, and 3.4 pg/mL troponin. Following the IV administration of 1 mg atropine, the pulse of the patient reached 100 beats per minute. The patient was discharged after undergoing monitoring for a certain period for normal sinus rhythm (Figure 4).

**Figure 5:**
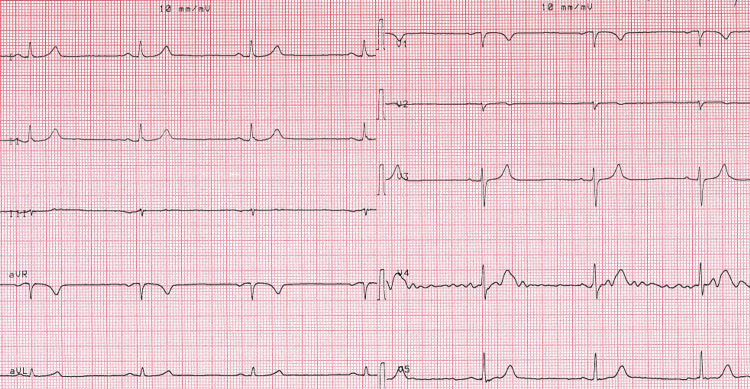
Sinus bradycardia before treatment.

**Figure 6:**
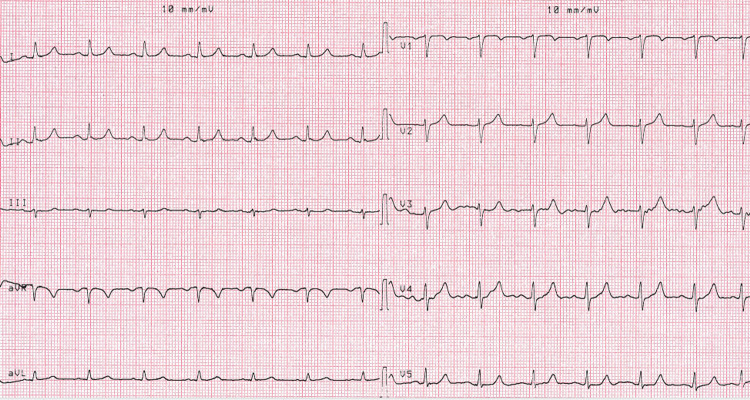
Sinus rhythm after IV administration of atropine.

Case 4: A 56-year-old male patient was admitted to our service following palpitation and a feeling of fainting after eating two teaspoons of wild honey. After the first physical examination, his pulse was found to be 41 beats per minute. Tension was 66/42 mm/hg. The laboratory findings were as follows: 9300 K/uL leukocytes, 13.4 g/dL hemoglobin, 271000 K/uL platelet, 19 U/L ALT, 19 U/L AST, <2.0 mg/L CRP, 37 mg/dL urea, 1.35 mg/dL creatinine, and 3.9 pg/mL troponin. The ECG findings were indicative of sinus bradycardia for the patient (Figure 5). Up to 1 mg atropine was intravenously administered to the patient, who exhibited a good response. In the next control, his pulse was 84 beats per minute, and the tension was 102/65 mmHg. The patient was discharged after a certain period of monitoring with a suggestion for a follow-up (Figure 6).

Case 5: A 50-year-old female patient was admitted to our service with symptoms of palpitation, dizziness, and bradycardia due to oral ingestion of mad honey (approximately 10 mL). The patient was normotensive (blood pressure 120/80 mm/Hg), her initial heart rate was 47 beats per minute and her SpO2 was 96%. The ECG findings were fully indicative of sinus bradycardia. The laboratory findings were as follows: 19 mg/dL urea, 0.72 mg/dL creatinine, 4.1 mmol/L potassium, 140 mmol/L sodium, 22 U/L AST, 25 U/L ALT, 6720 K/uL leukocytes, 12.5 g/dL hemoglobin, 241000 K/uL platelet count, 7.5 mg/L CRP, 20 U/L CK-MB, and 0.6 pg/mL troponin. Following the IV administration of 1 mg atropine, the pulse of the patient reached 85 beats per minute. The patient was discharged after undergoing monitoring for a certain period for normal sinus rhythm.

## Discussion

Mad honey, which is found in various types of rhododendron plants in the Eastern Black Sea region of Turkey, has been used for many years by the locals to treat diabetes, gastroduodenal maladies, and sexual dysfunction [3]. However, excessive consumption of this product can cause toxicity [4]. After the ingestion of wild honey, side effects such as exhaustion, dizziness, sweating, nausea, and vomiting occur. Apart from these side effects, shock, sinus bradycardia, nodal rhythm, Wolff-Parkinson-White syndrome, and complete block may ensue [5]. All five patients in this case series manifested exhaustion, dizziness, hypotension, syncope, and bradycardia. Grayanotoxin, which is responsible for mad honey poisoning, causes cardiac toxicity and sinus node dysfunction by increasing the permeability of the sodium channels [6]. Cases from Nepal and Turkey show that IV atropine provides a good response rate for cardiac side effects [7,8]. In three of our cases, good response to atropine was recorded, with the patients reverting into a normal rhythm after bradycardia. In Nepal, 15 cases were recorded between 2004 and 2012, eight of whom had sinus bradycardia, four had junctional bradycardia, two had complete AV block, and one had atrial fibrillation. Nearly all of the cases demonstrated a good response to atropine, similar to our four cases [9]. In the case series presented by Gunduz A et al., eight patients were evaluated. Among them, six were discharged following some period of monitoring. Two of the patients were monitored at the coronary ICU and a temporary pacemaker was attached to one of the patients [10]. Similarly, in one of our cases, no response to atropine was recorded. The patient was then transferred to the coronary ICU for monitoring and a temporary pacemaker was attached.

The toxic effects of honey can be dose-dependent, but the dose-response relationship was not evaluated conclusively; further research is indubitably warranted [4].

## Conclusions

On a final note, it is worth mentioning that mad honey can be found in various regions of the world, including Turkey. Its potential effects, especially its vital cardiac side effects, should be taken into account for patients with bradycardia who are admitted for emergency service.

## References

[1] Eroğlu SE, Urgan O, Onur OE, Denizbaşı A, Akoğlu H (2013). Grayanotoxin (mad honey) - ongoing consumption after poisoning. Balkan Med J.

[2] Ceter T, Guney K (2011). Ormangülü ve deli bal [Turkish]. Uludağ Arıcılık Derg.

[3] Jansen SA, Kleerekooper I, Hofman ZL, Kappen IF, Stary-Weinzinger A, van der Heyden MA (2012). Grayanotoxin poisoning: 'mad honey disease' and beyond. Cardiovasc Toxicol.

[4] Shrestha TM, Nepal G, Shing YK, Shrestha L (2018). Cardiovascular, psychiatric, and neurological phenomena seen in mad honey disease: a clinical case report. Clin Case Rep.

[5] Ergun K, Tufekcioglu O, Aras D, Korkmaz S, Pehlivan S (2005). A rare cause of atrioventricular block: Mad Honey intoxication. Int J Cardiol.

[6] Choo YK, Kang HY, Lim SH (2008). Cardiac problems in mad-honey intoxication. Circ J.

[7] Dubey L, Maskey A, Regmi S (2009). Bradycardia and severe hypotension caused by wild honey poisoning. Hellenic J Cardiol.

[8] von Malottki K, Wiechmann HW (1996). [Acute life-threatening bradycardia: food poisoning by Turkish wild honey]. Dtsch Med Wochenschr.

[9] Sohn CH, Seo DW, Ryoo SM, Lee JH, Kim WY, Lim KS, Oh BJ (2014). Clinical characteristics and outcomes of patients with grayanotoxin poisoning after the ingestion of mad honey from Nepal. Intern Emerg Med.

[10] Gunduz A, Turedi S, Uzun H, Topbas M (2006). Mad honey poisoning. Am J Emerg Med.

